# Neuroprotective and Nerve Regenerative Approaches for Treatment of Erectile Dysfunction after Cavernous Nerve Injury

**DOI:** 10.3390/ijms18081794

**Published:** 2017-08-18

**Authors:** Jeffrey D. Campbell, Arthur L. Burnett

**Affiliations:** Department of Urology, The James Buchanan Brady Urological Institute, Johns Hopkins Medical Institutions, Baltimore, MD 21287, USA; jcampb84@jhmi.edu

**Keywords:** erectile dysfunction, cavernous nerve injury, regenerative medicine, neuroprotection

## Abstract

Erectile dysfunction (ED) is a significant cause of reduced quality of life in men and their partners. Cavernous nerve injury (CNI) during pelvic surgery results in ED in greater than 50% of patients, regardless of additional patient factors. ED related to CNI is difficult to treat and typically poorly responsive to first- and second-line therapeutic options. Recently, a significant amount of research has been devoted to exploring neuroprotective and neuroregenerative approaches to salvage erectile function in patients with CNI. In addition, therapeutic options such as neuregulins, immunophilin ligands, gene therapy, stem cell therapy and novel surgical strategies, have shown benefit in pre-clinical, and limited clinical studies. In the era of personalized medicine, these new therapeutic technologies will be the future of ED treatment and are described in this review.

## 1. Introduction

Erectile dysfunction (ED) is the recurrent and persistent inability to achieve and maintain an erection adequate to permit sexual performance for at least 3 months [1]. ED is common worldwide and affects more than 50% of men between the ages of 40 and 70 years [2]. A 2004 prevalence study that included patients from North America, the United Kingdom, and Australia predicted that by 2025, more than 322 million men worldwide will suffer from ED [3,4]. More recently, the European Male Ageing Study (EMAS) reported a prevalence of 30–64% and approximately one-third of men in their population were dissatisfied with their overall sexual relationship [5].

Current therapeutic approaches most commonly involve oral therapies in the form of phosphodiesterase type 5 inhibitors (PDE5i). In addition, second-line alternatives such as intracavernosal injections (ICI) with vasoactive agents, vacuum erection devices, and third-line surgical approaches using inflatable penile prostheses offer ED management with varying results in quality of life (QOL) and functional outcomes [1]. Specific populations which are difficult to treat include those men who have undergone a radical prostatectomy (RP), have comorbid diseases such as diabetes mellitus (DM), and the aging population. Patient-reported outcomes after RP were recently reviewed in the ProtecT trial and sexual dysfunction was found to significantly impact health-related quality of life (HRQOL) in patients with prostate cancer [6,7]. Treatments for ED in post-RP patients have inconsistent safety and efficacy profiles, and there remains an ongoing search for a well-tolerated and clinically durable therapeutic option for treatment-refractory men [8,9].

The cavernous nerves (CN) are the principal autonomic innervation of the penis. The CNs originate from the pelvic ganglia (PG) and contain both sympathetic and parasympathetic nerve fibers. These nerves are almost exclusively unmyelinated and release the neurotransmitters for penile innervation [10,11]. Pelvic surgery, specifically RP and radical cystoprostatectomy, often results in a disruption of these nerves, leading to ED in greater than 50% of patients [12,13]. Even with bilateral nerve-sparing and minimally invasive approaches to RP, some degree of CN injury (CNI) or neuropraxia is inevitable. Advances in technology and the understanding of penile neurobiology have led to neuromodulatory strategies targeting CN protection and regeneration [8].

**Figure 1:**
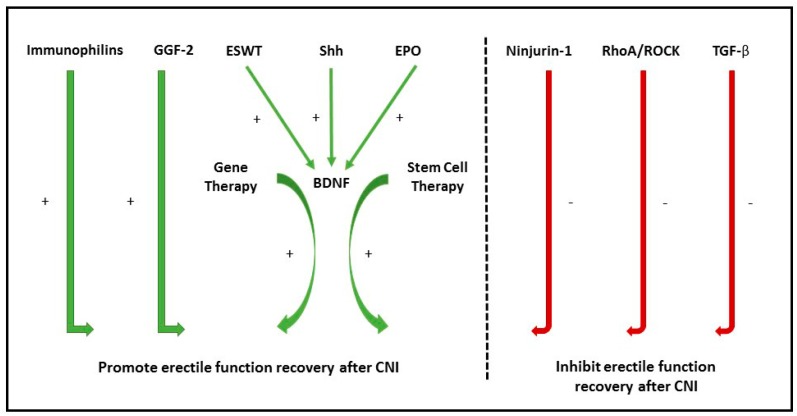
Summary of promoters and inhibitors of erectile function recovery after cavernous nerve injury (CNI). GGF-2: Glial Growth factor 2; ESWT: Extracorporeal Shock Wave Therapy; Shh: Sonic Hedgehog protein; EPO: Erythropoietin; BDNF: Brain-derived Neurotrophic Factor; Ninjurin-1: Nerve Injury-Induced Protein 1; ROCK: Rho-associated protein kinase; TGF-β: Transforming Growth Factor-β.

Due to the significant burden of disease, a copious amount of research has been focused on discovering the molecular pathways underlying neuronal control of erectile function, the mechanism of CNI, and novel therapeutic approaches that promote CN function recovery [8,14,15,16,17,18]. With recent advancements in our understanding of CNI and recovery, we move toward new therapeutic options for ED in treatment-refractory men [8]. Currently, there are no established treatment options that offer CN protection or regeneration at the clinical level, however, investigative efforts are ongoing. The following review provides an update on current research regarding the neuronal control of erectile function and how this correlates with translational advancement. We discuss molecular pathway targets that promote nerve regeneration and inhibitory pathways that need further regulation to prevent the development of post-CNI ED (Figure 1).

## 2. Neurophysiology of Erection

**Figure 2:**
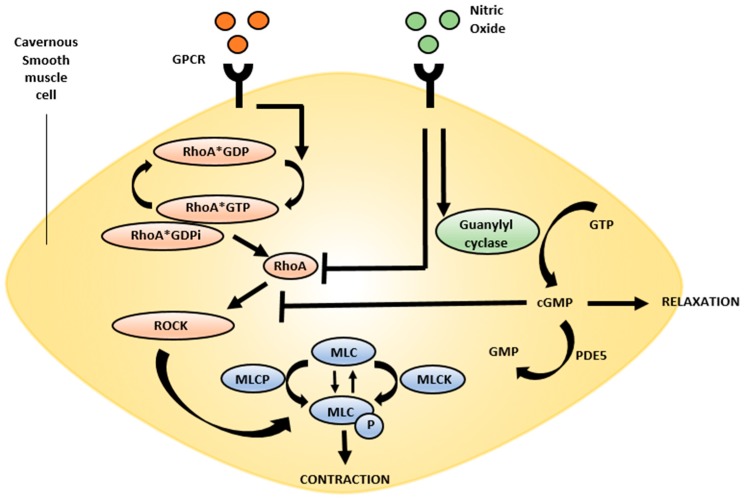
RhoA/ROCK pathway: Ligand binding to G-protein coupled receptors results in conversion of RhoA-GTP to RhoA-GDP. RhoA-GTP dissociates from RhoA-GDPi, allowing RhoA to bind downstream targets, including ROCK. ROCK autophosphorylates to become active and subsequently phosphorylates MLCP, rendering it inactive and promoting smooth muscle contraction and maintenance of the flaccid penile state. NO promotes the conversion of GTP to cGMP by activating guanylyl cyclase. Both NO and cGMP can inhibit the activity of RhoA thereby promoting penile relaxation. GTP: guanosine-5′-triphoshate; GDP: guanosine-diphosphate; GDPi: GDP dissociation inhibitor; ROCK: Rho-associated protein kinase; MCLP: myosin light chain phosphatase; NO: Nitric oxide; cGMP: cyclic guanosine monophosphate; GPCR: G Protein Coupled Receptor.

The neurophysiology of erection has been described for many years and its investigation has led to the advent of PDE5i for the treatment of ED. In summary, the neurotransmitter nitric oxide (NO) and the constitutive NO synthase (NOS) isoforms are the primary mediators of penile erection [19]. Both neuronal NOS (nNOS) found in nerve terminals and endothelial NOS (eNOS) found in vascular and sinusoid endothelium initiate and maintain penile erection. After phosphorylation, the constitutive forms of NOS are activated and generate NO from L-arginine [20,21]. After NO is produced, it diffuses into local smooth muscle tissue and binds to guanylate cyclase, which converts guanosine-5′-triphosphate (GTP) to cyclic guanosine monophosphate (cGMP). Protein kinase (PK) G is activated by cGMP and promotes corpus cavernosal smooth muscle relaxation, which results in penile erection (Figure 2) [20,22]. Erection is terminated when phosphodiesterase type 5 (PDE5) hydrolyzes the 3′5′ bonds of cGMP to render it inactive as 5′-GMP [23,24].

## 3. Nerve Regeneration Pathways in ED

Medical strategies for nerve protection during surgery or nerve regeneration post-operatively have been a recent focus of ED research. Next, we describe novel nerve regenerative targets which are being explored in neurologic ED and will likely have a significant future impact as therapeutic options.

### 3.1. Neurotrophins

Neurotrophins are a family of proteins that induce the survival, development, and function of neurons. Brain-derived nerve growth factor (BDNF), glial cell line-derived neurotrophic factor (GDNF), neurotrophin-3 (NT-3), and growth differentiation factor-5 (GDF-5) are neurotrophins that have demonstrated the ability to restore erectile function in animal models of ED [25,26,27]. After CNI, BDNF facilitates recovery of nNOS-containing nerve fibers and myelination of Schwann cells and thereby preserves erectile function [28,29,30]. Chen et al. studied the effect of intracavernosally injected neurotrophins in a CNI rat model. Rats injected with BDNF or vascular endothelial growth factor (VEGF) had improved erectile function compared to controls and their responses were heightened by the combination of these neurotrophins [26]. In a rat model of CNI, Bella et al. used inhibitors of different molecular pathways combined with BDNF, and demonstrated that Janus kinase/signal transducer and activator of transcription (JAK/STAT) is the major signal-transduction pathway of BDNF-enhanced CN fiber growth [31]. 

A recent investigation of the molecular mechanism of extracorporeal shock wave therapy (ESWT) for ED demonstrated an increase in BDNF after treatment [32]. The effect of ESWT on BDNF expression was found to work through activation of the PKR-like endoplasmic reticulum kinase (PERK)/activating transcription factor 4 (ATF4) signaling pathway [32]. This innovative finding offers a new mechanism for a local upsurge in a neurotrophin that has already demonstrated significant neuronal regenerative effects. The results of early work investigating BDNF in CNI models are promising, but further work in the field is required to optimize the clinical administration and utilization of this neurotrophin.

### 3.2. Sonic Hegdehog Protein

The hedgehog family consists of sonic hedgehog (Shh), desert hedgehog and Indian hedgehog proteins. Shh, the most studied of these proteins, is involved in cell differentiation of embryonic cells and plays an important role in regulating vertebrate organogenesis [33]. Shh is upregulated in mature cells after nerve injury and therefore is postulated to play a role in nerve regeneration [34]. Shh is abundant in the Schwann cells of CN and appears necessary for maintenance of CN morphology, although its mechanism of action is incompletely understood. Shh is produced within the CN nerve cells and has signaling to nearby glial cells [35]. Inhibition of this protein causes demyelination and axonal degeneration of CN fibers [36,37].

Over the past decade, Shh has been implicated in promoting CN regeneration in post-RP and diabetic conditions [35,38,39]. In DM animal models with evidence of neuropathy, Shh has been shown to promote nerve regeneration and decrease or prevent CNI-induced apoptosis [39,40,41]. Angeloni et al. applied Shh to CNI animal models with ED and demonstrated CN regeneration, suppressed penile apoptosis and improved erectile function [37]. Follow-up studies confirmed these results and employed techniques such as nanofiber hydrogel delivery to prolong the survival of Shh in CN. Treatment with nanofibers not only prevented neuronal degeneration, but maintains neuronal, glial and downstream target signaling, suggesting that this is a viable treatment option for regenerative medicine after CNI [42,43].

Animal models of CNI treated with Shh for two days demonstrate a 44% increase in BDNF expression [38]. It appears that the Shh pathway works symbiotically with BDNF to allow antegrade and retrograde signaling to prevent degeneration of nNOS-containing neurons in the pelvic ganglia and facilitate the regeneration of nNOS-containing fibers in penile tissue [38]. This intriguing innovation provides an avenue to explore combination therapy that can manipulate CN signaling, promote nerve regeneration, and salvage erectile function after CNI.

### 3.3. Ninjurin-1

Nerve Injury-Induced Protein 1 (Ninjurin-1) is related to vascular regression during embryonic development, has inflammatory properties, and is predominantly expressed by monocytes [44]. Blockade of Ninjurin-1 expression reduces myeloid cell recruitment during inflammation, and decreases infiltration of macrophages, dendritic cells, and antigen presenting cells (APC) into the central nervous system [45].

Ninjurin-1 has been explored as a therapeutic target for ED related to CNI. Local treatment with the monoclonal neutralizing antibody (Ninj-1mAb) was found to increase penile nNOS, decrease endothelial cell apoptosis, encourage cavernous endothelial proliferation, and induce phosphorylation of eNOS and PKB in the penis [46]. CNI rat models treated with high-dose Ninj-1mAb had a greater than 90% recovery of erectile function compared to controls [46]. In a DM rat model of ED, Ninjurin-1 blockade facilitates penile angiogenesis and neural regeneration, ultimately resulting in recovery of erectile function [47]. The dual neurotrophic and angiogenic effects of Ninjurin-1 blockade provide an opportunity for treating ED related to CNI [46]. Studies promoting a blockade of these inflammatory mechanisms induced by CNI are underway and harbor the potential for developing a molecular regenerative approach to treat ED in patients with neurogenic ED.

### 3.4. Neuregulins

Neuregulins represent a family of growth factors that function by binding ErbB tyrosine kinase transmembrane receptors [48]. These growth factors stimulate cell proliferation, differentiation, and cell survival and play a crucial role in axoglial signaling during the development of the peripheral nervous system [48]. Additionally, neuregulins mediate signals between axons and Schwann cells and play a key reparative role in adult nerve injury [49].

Glial growth factor-2 (GGF-2) is a member of the neuregulin family that has neuroprotective and neurorestorative properties. GGF-2 is encoded by the *Neuregulin-1* gene and is type 2 of at least 6 known variants. Neuregulins have been studied in many disease processes and due to their regenerative mechanism in nerve injury, they have recently been investigated for their potential role in neurogenic ED. GGF-2 was first explored in a CNI rat model by Burnett et al. in 2015 [50]. After inducing a bilateral CNI, GGF-2 protein was administered intracavernosally once weekly for 5 weeks. Erectile function was evaluated in the study groups by CN stimulation. Treatment with GGF-2 preserved unmyelinated CN fibers, sustained axonal integrity, and promoted erectile function recovery after CNI [50]. This early study provides some insight into the role of neuregulins in the treatment of ED, although further pre-clinical studies are necessary before expanding to human trials.

### 3.5. Immunophilins

Immunophilins are *cis*–*trans* peptidyl-prolyl isomerases that were originally identified as receptor proteins that bind and mediate the immunosuppressive effects of drugs such as cyclosporin, tacrolimus (FK506), and rapamycin [51]. The two main immunophilin families are the cyclosporin-binding cyclophilins (CyPs) and the FK506-binding proteins (FKBPs) [52]. FKBPs act as chaperone proteins by regulating protein folding, and participating in intracellular protein trafficking [53]. In addition to their role in the immune system, immunophilins are abundantly present in the central nervous system and have nearly identical regional localization to calcineurin [54]. Immunophilins have demonstrated neuroregenerative effects in rat models. Using ischemic stroke and peripheral nerve injury animal models, treatment with FK506 has been shown to prevent nerve injury and enhance functional recovery [55,56,57]. The mechanism of neuroprotection by immunophilins is incompletely understood.

The first study of immunophilins in CNI rat models was performed in 2001 and utilized FK506 [58]. Erectile responses were measured at multiple time points after CNI and rats treated with FK506 demonstrated improved penile pressures. In addition, penile tissue of FK506 treated rats revealed an increase in the number of surviving unmyelinated axons [58]. A follow-up study confirmed the neuroregenerative benefit of FK506 by using a complete CNI model [59]. These studies demonstrate a clear improvement in erectile function in animal models treated with FK506.

## 4. New Molecular Targets for Neurogenic ED

Neurovascular homeostasis is essential for production and maintenance of penile erection. A fine balance between NO, guanylate cyclase and PDE5 activity is critical for local concentrations of cGMP and smooth muscle relaxation. After CNI, there is an imbalance of the NO/CGMP pathway which clinically results in ED. New molecular markers have been recognized to preserve homeostasis through alternate pathways and are reviewed here.

### 4.1. Rho Kinase Pathway

RhoA is a small monomeric member of the Ras-GTPase family and is a key intracellular regulator involved in controlling actin-myosin contraction through activation of Rho-associated protein kinase (ROCK). Ligand binding of smooth muscle G-protein coupled receptor (GPCR) promotes the conversion of RhoA-GDP to RhoA-GTP. Next, RhoA-GTP dissociates from the RhoA-GDP dissociation inhibitor, which enables RhoA to migrate to the cellular membrane and bind other targets, including ROCK [60,61,62]. ROCK subsequently phosphorylates myosin light chain phosphatase (MLCP), which renders it inactive. This process sensitizes the myosin-actin contraction to lower levels of cytosolic calcium in smooth muscles, which facilitates tonic contraction and the penile flaccid state [61,62,63]. In summary, RhoA and ROCK are required for penile detumescence and flaccidity through their inhibition of MLCP and subsequent promotion of penile smooth muscle contraction (Figure 2).

Although controversial, there does appear to be interplay between the NO and RhoA pathways. Originally investigated in hypertension models, activation of the NO pathway was found to inhibit RhoA-mediated smooth muscle contraction [64]. In corporal smooth muscle, this mechanism results in an amplification of the erectile response. On the contrary, chronic inhibition of NO is associated with decreased RhoA activity. Stimulation of the NO pathway in these models results in increased RhoA protein expression and stabilization [65]. It is believed that operating together NO and RhoA maintain vascular homeostasis between the relaxed and contractile states [61]. Further work exploring the precise molecular mode of action and complex interplay of these proteins is needed to elucidate a specific clinical target.

The RhoA/ROCK pathway also plays an important NO-independent role in regulating smooth muscle tone in penile tissues [66]. After injury to peripheral nerves, increased activity of the Rho/ROCK pathway is observed [67]. After CNI, membrane bound RhoA and ROCK activity increases in the corporal endothelial and smooth muscle cells of the penis. Activation of this pathway subsequently leads to a decrease in nNOS and eNOS expression as well as corporal smooth muscle cell apoptosis [61]. ROCK2 expression is upregulated in CNI-related ED, whereas ROCK1 is overexpressed in DM-induced ED [61,68]. In animal studies, inhibition of the RhoA/ROCK pathway results in axonal regeneration, decreased cellular apoptosis, and prevention of tissue fibrosis, both systemically and locally in the penis [69,70,71].

### 4.2. Transforming Growth Factor-β

Recent attention to inflammatory and fibrotic cascades in the penis after RP have necessitated exploration of mediators to prevent long-term complications such as ED. Transforming growth factor-β (TGF-β) has been demonstrated to be elevated after CNI and is implicated in post-RP fibrosis. Histone deacetylase (HDAC) controls gene expression by catalyzing the hydrolysis of acetyl-l-lysine side chains in histone and non-histone proteins [72]. The enzymatic activity of HDAC plays a role in the development and progression of fibrosis in chronic inflammatory states through gene transcription of key profibrotic proteins [73]. A strict balance between histone acetyltransferase (HAT) and HDAC must exist to regulate transcription of these mediators. Excessive activation of HDAC induces inflammation, cell proliferation and fibrosis, which is pathologic in many disease process, including ED. HDACs are required for activation of the extracellular signal-related kinase (ERK) and phosphoinositide-3 kinase (PI3K) signaling pathways by TGF-β and the subsequent profibrotic genes dependent on these signaling pathways [74]. TGF-β nullifies HDAC deacetylation, which leads to vascular remodeling and fibrosis. Immunohistochemical analysis of penile tissue of rats after CNI demonstrates an increase in penile HDACs and TGF-β1 at 14 days, with associated penile fibrosis and regression of erectile function [75].

### 4.3. Erythropoietin

Erythropoiesis, the production of red blood cells, is under strict control by the cytokine erythropoietin (EPO). The receptor for EPO is abundantly present within both central and peripheral nervous systems and plays a role in neural degenerative diseases by attenuating hypoxic insults [76,77]. Recombinant EPO has been successfully used in clinical trials to improve neurologic function following ischemic stroke in humans [78,79]. The neurotrophic properties of EPO make its use particularly attractive for CNI models of ED. EPO therapy has a molecular basis that appears to involve neurotrophic transcriptional factors and neuronal cell survival kinase mechanisms. Combined, these neurologic molecular pathways exert anti-apoptotic effects and activate BDNF, which we know has a protective role in neurogenic ED [80].

EPO was first explored as a possible treatment option for sexual dysfunction in patients with end-stage renal disease on hemodialysis (HD). The initial study looked at the penile Doppler of 6 men on HD and compared arterial flow before and after EPO administration [81]. There was a significant improvement in brachial indices, shaft circumference, penile length, and penile angle, which indicates an increase in vascular flow. The results of this study were not statistically significant given that only 6 patients were studied [81]. However, this did prompt further investigation into the molecular cause of improved erectile function with EPO, beyond simply the correction of anemia.

EPO receptors have been discovered within the urogenital tract, and specifically within the neurovascular bundles, prostate epithelium, penile dorsal nerves, sinusoids of the corpus cavernosum, and the endothelial lining of penile vasculature [82,83]. Given the wealth of receptors in erectile tissue, it was hypothesized that the neuromodulatory effect of EPO is translatable for the treatment of neurogenic ED. Animal models of CNI have been used to explore EPO as a treatment option. CNI rats treated with recombinant human EPO effectively recovered erectile function without significant complication and demonstrated significant CN axonal regeneration [83].

## 5. Therapeutic Strategies for Neurogenic ED

Several approaches have been postulated to explore the proposed mechanisms of neural protection and regeneration. These ideas are currently still under investigation but do pose some interesting perspectives and promising therapeutic options. Limited clinical trials have been completed and are reviewed along with pre-clinical studies to date.

### 5.1. Tacrolimus (FK506)

For ED secondary to CNI, tacrolimus and similar immunophilin ligands, offer the advantage of being lipophilic, non-peptides that are selectively effective for damaged nerves [53]. Analogs of tacrolimus, including GPI-1046, GPI-1485, and FK1706 were developed and tested in neurogenic ED to circumvent the adverse immunosuppressive and toxic effects of chronic immunophilin administration [53,84,85]. The non-immunosuppressive immunophilin ligand GPI-1046 preserves erectile function and conserves CN axonal viability after CNI in rats [84]. GPI-1046 has been identified as a potential therapeutic opportunity for treatment of ED related to CNI, especially in patients who have undergone an RP. Moreover, a phase II multi-center, randomized, double-blind, placebo-controlled trial was conducted to evaluate GPI-1485 in men who have undergone nerve-sparing RP. This well-conducted study did not reveal any significance among treatment groups, but further studies with this ligand should be developed to optimize drug dosing strategies [86].

Although FK506 has successfully improved erectile function in animal models of CNI, an unpublished clinical trial failed to show a significant improvement in humans. In 2010, Mullhall et al. presented their findings of a Phase IV clinical trial at the American Urological Association meeting [87]. In this randomized, multi-center, placebo-controlled trial, study patients were treated with tacrolimus (Prograf^®^) for 6 months after RP and followed for two years. There was no significant improvement in erectile function after treatment and there were notable side effects from long-term use of tacrolimus including, renal toxicity, neurotoxicity, gastrointestinal upset, and electrolyte abnormalities [87]. Since the presentation of these results, research on immunophilin therapy for RP induced ED has ceased. Based on the animal studies already presented, more opportunities appear to be available as there are still significant gaps in our knowledge and understanding of these neuroprotective proteins. Multiple molecular pathways may be targeted for recovering erectile function after CNI and further development of more selective immunophilin ligands is warranted.

### 5.2. Rho-Kinase/ROCK Inhibitors

Inhibition of the Rho/ROCK pathway is an intriguing consideration for the treatment of neurogenic ED. Both pre-clinical animal studies and early clinical trials have targeted this pathway. When administered intracavernosally in a rat model of CNI, the non-selective ROCK inhibitor, Y-27632 (Tocris Bioscience, Ellisville, MO, USA) significantly decreased RhoA expression, increased nNOS expression, and improved erectile function [68]. Similarly, intraperitoneal administration of Y-27632 restored erectile function by day 14 in a CNI rat model. Treatment with this drug has demonstrated an increase in nNOS and eNOS expression, a decrease in ROCK activity, and a reduction in cellular apoptosis [62]. These animal studies did confirm that CNI upregulates the RhoA/ROCK signaling pathway, although subsequent inhibition of this pathway does salvage erectile response [62,68].

The encouraging results from the CNI rat models prompted a study utilizing human tissue extracted during penile prosthesis insertion. In patients with ED, ROCK2 was significantly elevated, corroborating the importance of this pathway in human pathology [88]. Treatment of preserved human tissue with Y-27632 demonstrated a significant relaxation of corporal cavernosum, which was additive when treated with vardenafil [88]. This innovative study using extracted human tissue may lead the way for clinical trials exploring Rho-kinase inhibitors as an oral monotherapy or combination therapy for treatment of neurogenic ED. The safety of Y-27632 needs to be evaluated after intracavernosal injection to penile tissue before this can be recommended for further use.

A more selective Rho-kinase inhibitor, SAR407899, is currently being evaluated clinically. The safety of SAR407899 was first evaluated in anti-hypertension studies, but subsequently has been proposed to treat ED in DM animal and human studies [89]. SAR407899 demonstrated an NO-independent benefit in erectile function in both models, which surpassed sildenafil [89]. Azaindole-1 is the newest selective ROCK inhibitor, which has demonstrated an improvement in erectile function in CNI rat models, but has not yet been evaluated in clinical trials [90]. Further studies need to be carried out in CNI models to explore the potential benefit of these inhibitors to improve erectile function in the absence of NO.

### 5.3. Valproic Acid

Valproic acid is a commonly prescribed anticonvulsant medication that acts through non-selective inhibition of HDAC. Systemic administration of this medication has been shown to reduce smooth muscle hypertrophy and TGF-β mediated fibrosis in hypertensive and optic nerve injury rat models [91,92]. In a novel study using the same anti-inflammatory premise, CNI rats were treated with valproic acid, which demonstrated a recovery of erectile function. Further evaluation has confirmed that valproic acid lowers penile HDAC and TGF-β1 expression, thus reducing penile fibrosis [75]. This original study offers a new method for neuroprotection that should be further evaluated for a potential use peri-operatively for men undergoing RP.

### 5.4. Erythropoietin

The first investigation of the clinical efficacy of EPO for the treatment of ED in the post-RP setting was done by Burnett’s group in 2008 [93]. This was a small retrospective study which used off-label EPO injections for treatment of ED after RP. Patients were given 40,000 IU of EPO preoperatively when undergoing nerve-sparing RP, after which they were evaluated with IIEF questionnaires. After 1-year follow-up, there was a higher percentage of men in the EPO group who were sexually active, but given the small numbers, this was not statistically significant. Importantly, a significantly higher proportion of patients in the EPO group reported clinically meaningful erections allowing for completion of sexual intercourse [93]. Prospective clinical trials exploring the benefit of peri-operative EPO need to be performed for further verification of the clinical benefit of this ubiquitous molecular pathway.

## 6. Regenerative Medicine for Neurogenic ED

Regenerative medicine is the concept of functional and structural restoration of diseased or damaged tissues and cells by replacement or stimulation of endogenous regenerative capacity. Restoring the expression of downregulated genes in erectile tissue and promoting nerve regeneration after CNI with stem cells and neurotrophic growth factors offer a huge opportunity for novel therapeutic strategies [14].

### 6.1. Gene Therapy

In the era of personalized medicine, gene therapy is becoming progressively more popular for treatment of many disease processes. Men who are refractory to PDE5i often have complex underlying pathophysiology. Most gene therapy in ED remains preclinical, however advances in our understanding of the molecular control of erectile function has led to progress in the field [94]. Summaries of pre-clinical gene therapy studies are reported elsewhere in detail and are beyond the scope of this review [14,95]. The potential clinical advantages of a gene transfer therapy for treatment of neurogenic ED include a durable, single therapy to restore erectile function, potential use in combination with other therapies to reduce dose requirements and side effects, and opportunity to develop patient-specific treatment approaches [96].

Gene therapy techniques to improve erectile function in the setting of CNI conceivable would focus on nerve regeneration through the novel pathways already discussed. Growth factors that have been explored as gene therapy for ED after CNI include: BDNF, GDNF, and neurturin [97]. BDNF and GDNF have been used in CNI animal models via adeno-associated and herpes simplex viral vector delivery, respectively [27,98,99]. Animal studies demonstrate an increase in intracavernosal pressure (ICP) after neuregulin gene therapy, however they do not achieve baseline erectile function [98,99].

To optimize the utilization of nerve regeneration growth factors, we need to explore the best, and safest vector, the growth factor with the least systemic side effects, and the best route of administration to augment erectile function. To date, the only ongoing clinical trials using gene therapy for treatment of ED are in DM patients [100,101]. No gene therapy models for treatment of CNI have made it to clinical trials, but based on the pre-clinical experiments there is a definite need for this endeavor.

### 6.2. Stem Cell Therapy

Stem cell therapy (SCT) is an emerging treatment option that has the potential to reverse the structural and neuronal causes of ED and mitigate patient dependence on the transitory effect of PDE5is. SCT is based on the ability of cells to differentiate into endothelial, nerve, and smooth muscle cells, thereby restoring normal tissue molecular signaling and architecture. Multiple types of SC have been used in animal models of ED. Adipose tissue-derived stem cells (ADSC), bone marrow-derived SC, and mesenchymal SC have successfully been investigated in CNI models of ED [102,103,104].

Bochinski et al. published the first report on the use of SCT in a rat model of CNI [105]. Embryonal SC from rat blastocysts were differentiated into neural cells by BDNF transfection and injected into the crus of the corpora or the pelvic ganglia. The treatment group had a significant improvement in erectile function and an increase in neurofilament staining [105]. Since this study, 28 additional pre-clinical trials have evaluated the use of SCT for regeneration of erectile function in CNI rat models and are reviewed extensively elsewhere [16,106,107,108]. The functional outcomes in the pre-clinical studies consistently demonstrate an improvement in erectile function [16,106]. Structural changes seen in the corpora cavernosum after SCT include an increase in endothelial and smooth muscle cell markers, an increase in neural cell markers, a decrease in collagen content, and a decrease in CN cell apoptosis [106]. Importantly, pre-clinical studies have established that treatment with intracavernosal SCT is safe in rats and acts as an adequate model which will prompt further investigation.

Recent advances in SCT research have prompted clinical trials in men post-RP. Haahr et al. obtained autologous ADSC from liposuction in 17 men with medically refractory ED after RP [109]. Intracavernosal injection of ADSC had no significant complications and demonstrated an improvement in IIEF scores [109]. Similarly, a recent study included 12 patients and used bone marrow-derived cells in post-RP patients to confirm safety of intracavernosally injected SC [110]. Phase II clinical trials with longer follow-up and more patients are needed to further evaluate this exciting application of SCT. Combining SCT with gene therapy or growth factors will almost certainly become a standard ED treatment in post-RP patients.

## 7. Surgical Strategies for Neuroprotection during Radical Prostatectomy

Although there have been significant improvements in the surgical technique of the RP, some degree of CNI does occurs, even with minimally invasive, bilateral nerve-sparing approaches [13]. CN interposition grafts have been evaluated during non-nerve sparing RP. Patients with high-grade disease typically require transection of NVBs in order to reduce the risk of a positive surgical margin and prevent poor oncological outcomes [111]. Sural and genitofemoral nerves have been used as interposition grafts in animal and human models [112,113,114,115]. Unfortunately, outcomes from nerve graft studies have been mixed and there is no clinical consensus regarding their benefit [111,116].

The most recent intraoperative strategy for nerve regeneration during nerve-sparing RP utilizes dehydrated human amnion/chorion membrane (dHACM) allograft nerve wraps (AmnioFix; MiMedx Group, Marietta, GA, USA) [117]. Studies using this technique evaluate its potential in patients with at least some degree of NVB preservation during robot-assisted laparoscopic RP (RALRP). The wrap is cut into strips and placed circumferentially around the NVB bilaterally, during extirpative RALRP, after urethral anastomosis [117]. Neurotrophic factors present in the dHACM promote CN cell survival and axon regeneration [118]. The mean time to recovery of potency and continence were both significantly shorter in the dHACM group, confirming some degree of neural regeneration [117,119]. Further studies using similar methodology is warranted to confirm the benefit of locally applied human amniotic and chorionic cells.

A novel strategy for CN protection during RALRP is regional hypothermia. Nerve inflammation due to cautery and mechanical trauma at the time of RP contributes to a delay in erectile function recovery. Finley et al. developed an endorectal cooling balloon, which they apply during RARP. Follow-up of their patients demonstrates a small, but statistically significant improvement in IIEF scores at 15 months [120,121,122,123]. Further studies utilizing hypothermia are needed to explore this novel neuroprotection strategy.

Finally, to minimize the degree of trauma to the NVBs, and promote post-operative nerve regeneration, CN stimulation during RP has been explored and optimization strategies are currently under development. The Cavermap^®^ surgical aid (Blue Torch Medical Technologies, Rockville, MD, USA) is the most studied CN stimulator. The stimulation probe is placed on tissue overlying the CN to elicit an erectile response to confirm nerve location. Unfortunately this technology has inconsistent results, low specificity, and a high false-positive rate, which limits its clinical use [124]. Additionally, nerve stimulators have shown a neuromodulatory benefit when applied to the peripheral nervous system [125,126]. Implantation of a nerve stimulator at the time of RP has therefore been proposed, not only for nerve identification, but for the postoperative regenerative benefits [127]. Further work exploring this opportunity is underway and offers a technology that may refine surgical technique and hasten the recovery of erectile function and continence after RP.

## 8. Conclusions

PDE5i have been the mainstay treatment of ED for years. Although studies have proven efficacy of these medications in certain populations, patients with CNI after pelvic surgery, or multiple medical comorbidities are more complex, rendering these medications less effective [128]. These difficult-to-treat patient populations have a poor QOL related to relationship and sexual dissatisfaction. New pathways including RhoA/ROCK signaling and penile fibrosis cascades offer alternative targets for intervention, which may be specific for CNI related ED. A significant amount of work has been done exploring both medical and surgical nerve regeneration strategies. Although limited clinical trials exist in the ED literature, these therapies are being utilized in other neurogenic pathologies and therefore offer hope for future use in urology. In the era of personalized medicine, understanding the neurologic pathways and novel growth factors involved in ED pathology will lead us to a realm of individualized gene and stem cell therapy. This is a very exciting time in ED research and we are optimistic that current methods to treat ED after CNI will soon be superseded by regenerative medicine.
